# Supplementary material for: Experiences of recently diagnosed urban COVID-19 outpatients: A survey on patient worries, provider-patient interactions, and neutralizing monoclonal antibody treatment
Source: PLoS One. 2025 Jun 23;20(6):e0325991. doi: 10.1371/journal.pone.0325991 (PMC12184928; doi:10.1371/journal.pone.0325991)
Supplement: S1 File — Additional methods including patient selection for creation of a survey eligibility cohort, sociodemographic variable definitions, and outcome questions. S1 Table. Adjusted odds ratio for trust in doctor for COVID-19 related care and treated fairly by doctors, demographic factors only. S2 Table. Adjusted odds ratio for worried about, difficulty getting, recommending, and never offered mAb treatment, demographic factors only. S3 Table. Adjusted odds ratio for worried about COVID-19 diagnosis, demographic factors only. (DOCX) [file pone.0325991.s001.docx]

**S1 File. Supplementary Methods**

**Patient Selection for Creation of a Survey Eligibility Cohort**

The biostatistics team was provided weekly with a cohort of adults who either tested positive for SARS-CoV-2 based on a PCR or antigen test or had a mAbs administration on or after November 1, 2020 from UCHealth electronic health record system. Patients who did not have a SARS-CoV-2 positive test date (COVID+ date) or mAb administration date (mAb date) were excluded. For patients missing COVID+ date with an observed mAb date, imputation of the missing COVID+ date was performed as follows. First, we computed the difference, in days, between mAb date and COVID+ date for patients who had both of these dates observed. For each patient with missing COVID+ date and observed mAb date, a random ‘difference’ was selected from this pool of differences and then the missing COVID+ date was imputed by subtracting the randomly selected difference from the patient’s mAb date. A population of individuals meeting inclusion criteria that had not previously been identified as survey eligible were identified weekly.

A survey selection date is defined as the date on which patients with SARS-CoV-2 infection, regardless of whether they received mAb treatment or not, were entered into the survey eligibility cohort. Before August 27, 2021, patients were eligible for selection into the survey eligible cohort if their COVID+ date was 90 days prior to the date of survey selection. On and after August 27, 2021, only patients who had COVID+ date within six weeks prior to the survey selection date, were alive and not hospitalized on or before their COVID+ date was eligible to be selected. If patients died before the survey selection process or were hospitalized on or before their SARS-CoV-2 positive test date, they were excluded from the study selection. Eligible patients were categorized into two groups based on their mAb treatment status, i.e., mAb-treated, and untreated groups. While all patients who received mAb therapy (mAb-treated patients) were selected for the survey eligibility cohort, we implemented a matching criterion based on propensity scores using nearest neighbor matching to select one to three untreated patients for each of the treated patient. Factors included in the logistic regression propensity model of mAbs were self-identified gender,, categorized age (18-54, 55-64, ≥65 years), self-identified race/ethnicity (non-Hispanic White, non-Hispanic Black, Hispanic, and others), whether the patient had conditions to fulfil the second FDA EUA eligibility criteria to receive mAb therapy, and number of days since SARS-CoV-2 positive test date up to the survey selection date (≤14, 15-28, and 29-42 days). From April 20, 2021 to July 22, 2021, a 10:1 matching ratio (i.e., 10 untreated patients for each treated patient) was performed. From August 27, 2021 to October 18, 2022 and from October 26, 2022 to December 28, 2021, a 3:1 matching and 1:1 matching, respectively, were carried out. Change in the untreated: treated ratio occurred to address non-response rates in the surveys over time.

**Survey Administration**

All survey completion occurred within 120 days of the positive SARS-CoV-2 test or mAb administration date with a minimum of 14 days between surveys. Participants were eligible for a single survey if enrollment and survey administration occurred more than 77 days after their positive SARS-CoV-2 test date. They were eligible for two surveys when enrollment and first survey administration occurred > 35 days and <= 77 days from their SARS-CoV-2 test date, and eligible for three surveys when enrollment and first survey administration occurred <=35 days from their SARS-CoV-2 test date.

**Sociodemographic Variable Definitions**

We categorized race/ethnicity into Non-Hispanic White, Non-Hispanic Black, Hispanic, and other (Asian, Native Hawaiian, Pacific Islander, American India, Alaskan Native, Other and Prefer not to answer responses); and age into three categories: 18-44, 45-64, and 65+ years old. When the sample size was small for some of our outcomes, we categorized race as Non-Hispanic White, Non-Hispanic Black/Other, and Hispanic. Immunocompromised status (yes/no) was based on a participant’s self-reported status regarding immune-suppressing medications, diagnosis of a health condition associated with a compromised immune system, and organ transplant status. Body mass index (BMI, in kg/m2) was dichotomized as a BMI <25 or >=25. We categorized the total number of other comorbid conditions (excluding immunocompromised and obesity status) as none, one, and two or more conditions. We obtained COVID-19 vaccination status from the state-wide Colorado Immunization Information System registry and linked it with the survey data and categorized as no vaccine, one vaccine, and two or more vaccinations at the time of SARS-CoV-2 positive test.

**Outcome Questions:**

The following questions were taken from the survey and used for this study:

*Q1. On a scale of 1 to 10, how worried were you when you learned you have COVID-19?*

*Q2. Do you think you were treated fairly when it comes to COVID-19-related care?*

*Q3. In general, how much do you trust that doctors or providers will do what is right when it comes to your COVID-19-related care?*

*Q4. How much do you trust that your doctor will do what is right when it comes to your COVID-19-related care?*

*Q5. To better understand why you did not receive the mAb treatment for COVID-19…do any of the following reasons apply to you? (asked only to those who did not receive mAbs; options for response included: it was never offered to me, it was offered but I didn’t want it, I was told I didn’t qualify, by the time I was going to get it I already felt better, by the time I was going to get it I was too sick, I had transportation problems, it was too complicated to figure out where to go, I was concerned about the cost of treatment, other reason, prefer not to answer))*

*Q6. On a scale of 1 to 10, how worried were you about getting the mAb treatment?*

*Q7. On a scale of 1 to 10, how difficult was it for you to get the mAb treatment?*

*Q8. If you had a close friend or relative who got COVID-19 and they were eligible to receive the mAb treatment…on a scale of 1 to 10, how likely would you be to recommend it?*

**Supplementary Tables**

**Supplemental Table 1. Adjusted Odds Ratio for Trust in Doctor to do What is Right for Your COVID-19 Related Care and Treated Fairly by Doctors, demographic factors only**

|  | Trust Doctor General | | Trust Main Doctor | | Treated Fairly | |
| --- | --- | --- | --- | --- | --- | --- |
|  | OR | 95% CI | OR | 95% CI | OR | 95% CI |
| **Age in Years** | | | | | | |
| 18-44 years | NA |  | NA |  | NA |  |
| 45-64 years | 0.79 | (0.56, 1.11) | 1.62 | (0.74, 3.55) | 0.84 | (0.54, 1.29) |
| 65+ years | 1.29 | (0.85, 1.97) | 2.83 | (1.12, 7.58) | 1.6 | (0.91, 2.85) |
| **Gender** | | | | | | |
| Male | NA |  | NA |  | NA |  |
| Female | 0.93 | (0.69, 1.26) | 0.81 | (0.40, 1.61) | 1.17 | (0.80, 1.72) |
| **Race/Ethnicity** | | | | | | |
| Non-Hispanic White | NA |  | NA |  | NA |  |
| Non-Hispanic Black/Other | 1.4 | (0.79, 2.63) | 4.95 | (0.98, 90.49) | 1.01 | (0.52, 2.12) |
| Hispanic | 1.09 | (0.73, 1.66) | 3.06 | (1.07, 11.33) | 0.95 | (0.58, 1.61) |
| **Level of Education** | | | | | | |
| < 2 yr college | NA |  | NA |  | NA |  |
| ≥ 2 yr college | 1.48 | (1.08, 2.02) | 2.57 | (1.22, 5.38) | 1.36 | (0.90, 2.05) |
| **Usual Place of Care** | | | | | | |
| Regular Doctor | NA |  | NA |  | NA |  |
| Walk-In Care | 0.53 | (0.37, 0.76) | 0.36 | (0.16, 0.90) | 0.6 | (0.39, 0.95) |
| **Health Insurance** | | | | | | |
| Commercial/Medicare | NA |  | NA |  | NA |  |
| Medicaid | 0.73 | (0.47, 1.14) | 0.82 | (0.32, 2.31) | 0.59 | (0.35, 1.01) |
| None/Other | 0.47 | (0.26, 0.85) | 0.9 | (0.21, 6.23) | 0.65 | (0.31, 1.53) |
| *Note:* |  |  |  |  |  |  |
| OR, odds ratio; CI, confidence interval |  |  |  |  |  |  |

**Supplemental Table 2. Adjusted Odds Ratio for Worried About, Difficulty Getting, recommending mAb Treatment and Never Offered mAb Treatment, demographic factors only**

|  | Received mAbs N=539 | | | | | | Didn't Receive mAbs N=986 | |
| --- | --- | --- | --- | --- | --- | --- | --- | --- |
|  | Worried Getting mAbs | | Difficulty Getting mAbs | | Recommend mAb | | Never Offered mAbs | |
|  | OR | 95% CI | OR | 95% CI | OR | 95% CI | OR | 95% CI |
| **Age in Years** | | | | | | | | |
| 18-44 years | NA |  | NA |  | NA |  | NA |  |
| 45-64 years | 0.73 | (0.39, 1.35) | 1.04 | (0.54, 2.09) | 1.03 | (0.42, 2.36) | 0.68 | (0.46, 1.00) |
| 65+ years | 0.55 | (0.29, 1.06) | 0.87 | (0.45, 1.74) | 1.39 | (0.56, 3.23) | 0.45 | (0.29, 0.69) |
| **Gender** | | | | | | | | |
| Male | NA |  | NA |  | NA |  | NA |  |
| Female | 2.09 | (1.30, 3.45) | 1.8 | (1.11, 2.96) | 0.78 | (0.42, 1.42) | 0.9 | (0.65, 1.24) |
| **Race/Ethnicity** | | | | | | | | |
| Non-Hispanic White | NA |  | NA |  | NA |  | NA |  |
| Non-Hispanic Black | 2.21 | (0.65, 6.99) | 1.56 | (0.33, 5.41) | 0.92 | (0.23, 6.31) | 0.85 | (0.45, 1.66) |
| Hispanic | 0.98 | (0.47, 1.92) | 0.55 | (0.20, 1.28) | 3.63 | (1.05, 22.96) | 1.18 | (0.74, 1.92) |
| Other | 0.7 | (0.03, 4.95) | 0.76 | (0.04, 4.94) | 0.51 | (0.07, 10.28) | 4.97 | (0.99, 90.44) |
| **Level of Education** | | | | | | | | |
| < 2 yr college | NA |  | NA |  | NA |  | NA |  |
| ≥ 2 yr college | 0.44 | (0.27, 0.72) | 1.03 | (0.60, 1.83) | 1.28 | (0.63, 2.48) | 0.76 | (0.53, 1.08) |
| **Usual Place of Care** | | | | | | | | |
| Regular Doctor | NA |  | NA |  | NA |  | NA |  |
| Walk-In Care | 0.57 | (0.26, 1.18) | 1.17 | (0.53, 2.36) | 2.71 | (0.90, 11.93) | 0.82 | (0.55, 1.22) |
| **Health Insurance** | | | | | | | | |
| Commercial/Medicare | NA |  | NA |  | NA |  | NA |  |
| Medicaid | 1.58 | (0.71, 3.39) | 0.23 | (0.04, 0.80) | 2.13 | (0.57, 14.03) | 1.79 | (1.02, 3.29) |
| None/Other | 3.23 | (0.62, 13.71) | 0.62 | (0.03, 3.71) | 0.6 | (0.09, 11.95) | 3.16 | (1.34, 9.33) |
| *Note:* |  |  |  |  |  |  |  |  |
| OR, odds ratio; CI, confidence interval |  |  |  |  |  |  |  |  |

**Supplemental Table 3. Adjusted Odds Ratio for Worried About Contracting COVID-19, demographic factors only**

|  | OR | 95% CI |
| --- | --- | --- |
| **Age in Years** | | |
| 18-44 years | NA |  |
| 45-64 years | 1 | (0.77, 1.30) |
| 65+ years | 0.74 | (0.56, 0.99) |
| **Gender** | | |
| Male | NA |  |
| Female | 1.82 | (1.47, 2.25) |
| **Race/Ethnicity** | | |
| Non-Hispanic White | NA |  |
| Non-Hispanic Black | 1.17 | (0.72, 1.89) |
| Hispanic | 1.57 | (1.14, 2.15) |
| Other | 2.39 | (0.92, 6.48) |
| **Level of Education** | | |
| < 2 yr college | NA |  |
| ≥ 2 yr college | 0.94 | (0.74, 1.19) |
| **Usual Place of Care** | | |
| Regular Doctor | NA |  |
| Walk-In Care | 0.91 | (0.69, 1.20) |
| **Health Insurance** | | |
| Commercial/Medicare | NA |  |
| Medicaid | 1.81 | (1.25, 2.62) |
| None/Other | 1.24 | (0.75, 2.07) |
| *Note:* |  |  |
| OR, odds ratio; CI, confidence interval |  |  |
