# Supplementary material for: Experiences of recently diagnosed urban COVID-19 outpatients: A survey on patient worries, provider-patient interactions, and neutralizing monoclonal antibody treatment
Source: PLoS One. 2025 Jun 23;20(6):e0325991. doi: 10.1371/journal.pone.0325991 (PMC12184928; doi:10.1371/journal.pone.0325991)
Supplement: Attachment — Submitted filename: PONE-D-24-35333 Review report.docx [file pone.0325991.s002.docx]

The manuscript (PONE-D-24-35333) offers a thorough summary of the study, encompassing its objectives, methodology, key findings, and conclusions. It effectively highlights the study's significance by connecting patient experiences, healthcare disparities, and treatment accessibility within the context of COVID-19. The study design is meticulously documented, providing clear details on participant selection, survey implementation, and statistical analyses. The manuscript may be accepted after major revision.

**Revisions Needed**:

1. Streamline content to emphasize critical findings (e.g., key socio-demographic factors).
2. Avoid redundant phrases like “Limited research has been performed...” which i
3. s already implicit in the introduction.
4. Include actionable insights to make the conclusion more impactful.
5. Condense background information to avoid over-explaining concepts such as monoclonal antibody (mAb) efficacy.
6. Clarify the novelty of the study by explicitly contrasting it with prior research.
7. Focus on the specific research gap that this study addresses.
8. Provide more detail on survey development, including pilot testing outcomes and psychometric validation.
9. Clarify the rationale for categorization and thresholds (e.g., Likert scale recategorization from 1-10 to 1-3, 4-6, and 7-10).
10. Discuss potential biases introduced by reliance on telephone surveys and verbal consent.
11. Improve clarity by summarizing findings for each outcome variable in dedicated subheadings (e.g., “Trust in Providers,” “Worry About COVID-19”).
12. Address potential confounding variables, particularly for outcomes related to mAb treatment and worry levels.
13. Enhance the discussion on why specific sociodemographic groups (e.g., vaccinated patients) reported higher worry levels.
14. Provide more actionable solutions to address disparities (e.g., interventions to improve trust in walk-in clinics or reduce barriers for Medicaid patients).
15. Address broader implications of findings, such as policy-level recommendations for equitable healthcare delivery.
16. Ensure all figures and tables are self-explanatory by adding detailed captions.
17. Simplify complex visualizations, particularly in Figures 1 and 2, to enhance readability.
18. Add comparative summaries in tables for quicker reference.
19. Edit for grammatical consistency and conciseness. For example, “Patients of younger age, female sex…” could be simplified to “Younger and female patients.”
20. Avoid repetitive phrases and overuse of technical jargon that could be streamlined for readability.
21. Use active voice where appropriate to improve flow (e.g., “We conducted a survey” instead of “A survey was conducted”).
22. Expand on how the urban-centric and English-speaking population limits the applicability of findings to diverse settings.
23. Address the limitation of self-reported data and potential underreporting or over-reporting biases.
24. Ensure all references are correctly formatted according to journal guidelines.
