# Supplementary material for: Experiences of recently diagnosed urban COVID-19 outpatients: A survey on patient worries, provider-patient interactions, and neutralizing monoclonal antibody treatment
Source: PLoS One. 2025 Jun 23;20(6):e0325991. doi: 10.1371/journal.pone.0325991 (PMC12184928; doi:10.1371/journal.pone.0325991)
Supplement: Attachment — Submitted filename: Acceptance letter to PONE-D-24-35333R1.docx [file pone.0325991.s004.docx]

The Editor
PLOS ONE

**Subject:** Recommendation for Acceptance of Manuscript (PONE-D-24-35333R1)

Dear Editor,

I am writing regarding the revised manuscript titled, *“Experiences of recently diagnosed urban COVID-19 outpatients: a survey on patient worries, provider-patient interactions and neutralizing monoclonal antibody treatment”* (Manuscript PONE-D-24-35333R1).

After carefully reviewing the authors’ responses to the comments during the initial review, I find that they have addressed the concerns comprehensively and have made the necessary revisions to strengthen their manuscript. Their responses were thorough, and the revised manuscript is now much improved.

The study provides significant insights into the experiences of COVID-19 outpatients and contributes valuable information on an important public health topic. Given the satisfactory revisions and the importance of the subject matter, I recommend that the manuscript be accepted for publication in *PLOS ONE*. Additionally look for the references style and other things to rule out any errors that are not in the preview of journal’s guidelines.

Thank you for the opportunity to review this manuscript.

Dr. Puneet K Singh
